# Long-term outcomes of lacrimal canalicular trephination with viscoelastic-assisted monocanalicular stenting for canalicular obstructions

**DOI:** 10.22336/rjo.2022.10

**Published:** 2022

**Authors:** Manpreet Singh, Manpreet Kaur, Zoramthara Zadeng, Manjula Sharma, Aditi Mehta, Pankaj Gupta

**Affiliations:** * Department of Ophthalmology, Advanced Eye Centre, Post Graduate Institute of Medical Education and Research, Chandigarh, India

**Keywords:** canalicular obstruction, lacrimal trephine, monocanalicular stent, punctum obstruction, viscoelastic

## Abstract

**Purpose:** To study the long-term outcomes of lacrimal canalicular trephination (LCT) with viscoelastic-assisted monocanalicular stenting (VAMS) for the treatment of epiphora secondary to lacrimal canalicular obstructions (LCO).

**Methods:** Our study was a retrospective interventional work. All patients diagnosed with LCO, having morbid epiphora (Munk’s scale ≥ 2), were included. The LCO was divided as proximal (< 6mm from punctum) and distal (≥ 6mm from punctum). Sisler’s lacrimal trephine (21 gauge) was used to recanalize the LCO with monocanalicular stent (0.64mm diameter) insertion, which was kept for a minimum of 6 weeks and a post-stent removal follow-up of 12 months was ensured. Fluorescein dye disappearance test and lacrimal irrigation were used as functional and anatomical tests for evaluation, respectively.

**Results:** We included 73 eyes of 52 patients having a mean age of 44.5 years. Of the total, the proximal LCO was seen in 38 eyes (52.1%) and distal in 35 eyes (47.9%). The preoperative Munk’s score of 5 was noted in the majority (n=57 eyes, 78.1%). The majority (n=32 eyes, 43.8%) had chronic blepharitis or meibomian gland disease as etiology. Monocanalicular stent was kept in place for a mean of 13.5 weeks. At a mean follow-up of 14.5 months, complete response was noted in 35.6% cases, while 50.7% had partial and 13.7% had a failure of the procedure.

**Conclusions:** LCT (without DCR) is a minimally invasive, simple, and effective technique for the treatment of LCO in the long term. VAMS is a helpful innovation to facilitate the insertion of the flexible silicone stent.

## Introduction

Lacrimal punctum and canaliculus constitute the proximal lacrimal drainage system (PLDS). An obstruction or stenosis of PLDS may cause clear fluid epiphora leading to a significant deterioration in the quality of life of patients [**1**]. The etiology of PLDS obstructions can be congenital, inflammatory (diseases, drugs, radiation), neoplastic, traumatic, and idiopathic [**2**]. Lacrimal canalicular obstruction (LCO) constitutes 0.92%-4.5% of epiphora patients [**3**,**4**].

The obstruction of PLDS is a complicated scenario due to unclear etiology of obstruction, unknown length of obstruction, and variable success rate of recanalization procedures depending upon the distance from the lacrimal punctum [**1**,**2**,**5**]. The available treatment options include a) conjunctivodacryocystorhinostomy (CDCR) + bypass tube, b) conjunctivo-rhinostomy + bypass conduit, c) dacryocystorhinostomy (DCR) + canalicular trephination with stenting, d) DCR + retrograde canalicular stenting recanalisation of obstruction, and e) canalicular trephination + monocanalicular stenting [**2**,**5**]. Most of the procedures mentioned above have limited popularity due to their tedious and time-consuming nature with frequent complications and variable success rates in different surgical hands [**2**,**5**].

Lacrimal canalicular trephination (LCT) with monocanalicular stenting is a simple and effective procedure for LCO [**1**,**5**]. Historically, Sisler and Allarakhia (1990) invented a lacrimal transcanalicular mini-trephine for treating LCO as an office-based procedure [**6**,**7**]. Its simple design and easy to use made Sisler’s lacrimal trephine one of the most favorite instruments in the armamentarium of a dacryologist [**1**,**5**,**8**]. This trephine has attracted worldwide attention and has been used with success rates ranging from 52-92% [**6**-**15**]. This technique was described in one of the pioneering articles in 2014 in patients of idiopathic lower LCO with a satisfactory success rate (83.3%) over 8.6 months [**8**].

To the best of our knowledge, the long-term outcomes of this procedure have still not been reported. Moreover, most studies featuring Sisler’s trephine have been conducted in post-DCR patients with or without nasal endoscopic guidance [**9**-**15**]. Hence, we planned to study the long-term outcomes of lacrimal canalicular trephination (LCT) with viscoelastic assisted monocanalicular stenting (VAMS) in patients with LCO.

## Methods

This is a single-institution, retrospective analysis of consecutively diagnosed patients having LCO, who presented to our Oculoplastics Clinic from Jan 2017 to December 2019 (3 years). Written informed consents were obtained from all patients of LCO, who underwent LCT using a Sisler’s lacrimal trephine (Beaver-Visitec, UK) with insertion of monocanalicular stent. All procedures were performed by a single surgeon (MS) in the main operating room under local anesthesia. We have obtained approval to conduct this retrospective study from our Institution’s review board. Proper informed consent was obtained from the participants in the procedure and publication of their data and unidentifiable clinical pictures in scientific journals. Our study adhered to the tenets/ guidelines laid by the declaration of Helsinki.

**Fig. 1:**
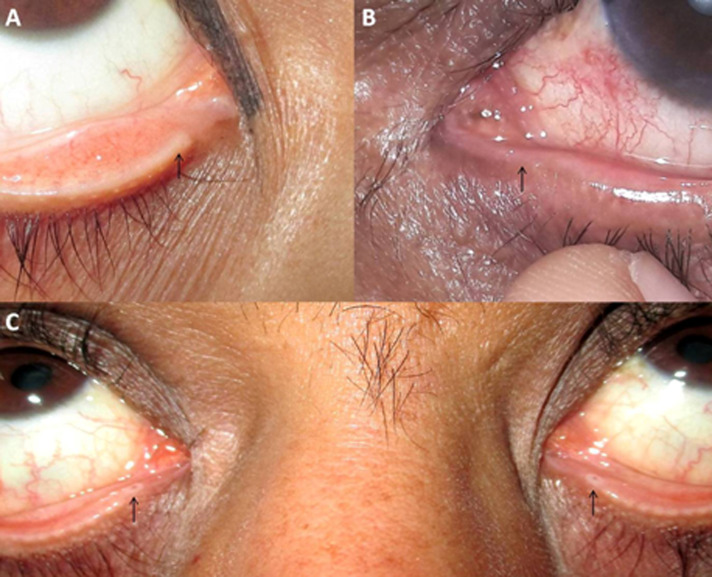
Clinical features of punctum and canalicular stenosis or obstruction. **A.** Right inferior punctum stenosis with hypotrophic canalicular region suggestive of canalicular stenosis or obstruction; **B.** Left inferior punctum and canalicular obstruction; **C.** Bilateral inferior punctum stenosis and distal canalicular obstruction with prominent peri-punctal fibrotic rings

The patients included had LCO causing clinically significant epiphora as per the Munk score of ≥ 2 [**16**]. Patients having a patent or stenosed punctum (**Fig. 1 A, B**) and proximal patent canaliculus of ≥ 2 mm from punctum were included in our study [**2**]. In patients having both upper and lower canalicular obstructions, the lower was chosen for the treatment in anticipation of better outcomes. ≥ 6mm from the lacrimal punctum was defined as distal (**Fig. 1 C**), and < 6mm from punctum was defined as proximal LCO. An obstruction located ≥ 10 mm from the lacrimal punctum, with clear fluid regurgitation from the opposite punctum, a history of eyelid malignancy causing LCO, congenital canalicular agenesis/ obstruction, acute conjunctivitis, traumatic canalicular laceration, previous lacrimal trephination, and eyelid malpositions were considered exclusion criteria. A minimum follow-up of 12 months after stent removal was considered an inclusion criterion.

Data collected included demographics, laterality, medical history (drugs, radiation, trauma), previous surgical treatment, Munk scoring for epiphora, cause/ location/ type of LCO, and treatment advised [16]. Clinical evaluation included a fluorescein dye disappearance test (FDDT) and lacrimal probing using Bowman’s lacrimal probe no. 0 for locating the level of LCO. The technique of measuring or finding the level of LCO has also been described by us [**1**,**8**]. 

*Surgical technique*

**Fig. 2:**
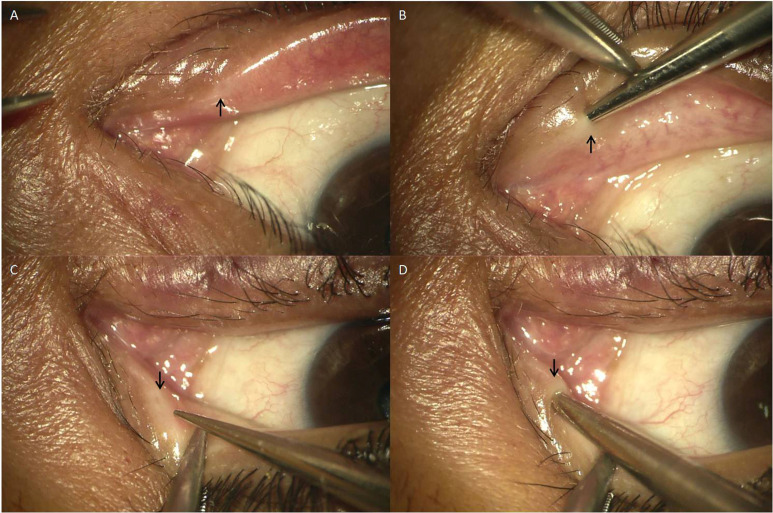
Location and dilation of stenosed puncta. **A.** Right inferior punctum stenosis (black arrow) and proximal canalicular obstruction; **B.** Punctum dilation done with Wilder’s sharp tip punctum dilator; **C.** Right superior punctum stenosis and proximal canalicular obstruction identified; **D.** Superior punctum dilatation done in similar way

**Fig. 3:**
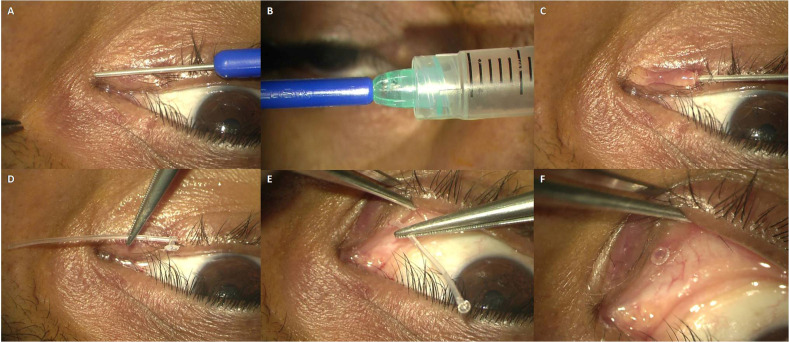
Lacrimal canalicular trephination and viscoelastic assisted monocanalicular stent insertion. **A.** Sisler’s lacrimal trephine and its planned path of insertion; **B.** Luer-lock at the distal end of Sisler’s trephine attached to a viscoelastic syringe; **C.** Exterior description of the viscoelastic layout inside the trephined tract; **D.** Monocanalicular stent with proximal punctum fixation device; **E.** Easy insertion of monocanalicular stent in the viscoelastic coated trephined canaliculus; **F.** After complete insertion of a stent, the collarette is seen lying flush with punctum in the desired way

The morphology and functioning of Sisler’s lacrimal trephine, monocanalicular stent, and the basic surgical procedure of lacrimal trephination have been described in the previous publications by the authors [**1**,**8**]. A proper lacrimal punctum dilatation with Nettelship/ Wilder’s punctum dilator (**Fig. 2**) was performed for atraumatic insertion of the probe or lacrimal trephine. The LCT was successfully done, and the patency of NLD was confirmed with irrigation, the viscoelastic syringe was attached to the distal end of Sisler’s trephine, having a luer-lock. At that moment, the dispersive viscoelastic was injected slowly while withdrawing the trephine from the canaliculus, laying the viscoelastic in a string-like fashion inside the newly trephined tract (**Fig. 3 A-C**). Undue compression over the eyelid was avoided to ensure the maximum retention of viscoelastic inside the canaliculus, and the patient was advised to look up for minimizing the orbicularis contraction. As a surgical innovation, we have used a viscoelastic agent to maintain the intraoperative patency and possibly “dilate” the trephined portion of the canaliculus. It facilitated the smoother passage of the monocanalicular stent (**Fig. 3 C-F**) via the narrow-trephined portion. The rest of all the steps were performed as described earlier [**1**,**8**]. 

Postoperatively, the patients were followed up on days 1, 7, 14, 4 weeks, 8 weeks, 12 weeks, 6 months, and 12 weeks. Topical moxifloxacin (0.5%) and dexamethasone (0.1%) combination was given for the first 2 weeks with carboxymethylcellulose 1% eye drops (QID). Patients were instructed not to rub their eyes and pull out the stent. In a few cases of “on-table” stent instability, a single suture with 10-0 nylon was applied to prevent the stent prolapse/ loss. The antibiotic-steroid combination eye drops were tapered weekly. The collarette of the stent was observed for its position and stent stability. The punctum and canalicular region were examined for any inflammation, discharge, or granuloma.

On each follow-up, 5 minutes FDDT was performed in all patients and was graded as negative, delayed, and positive. In all patients, the monocanalicular stents were kept for a minimum of 12 weeks, and stent removal was planned on subsequent follow-up visits. The outcomes were defined as complete response, partial response, and failure. 

• Complete response = complete relief from epiphora (Munk’s grade 0-1), negative FDDT, and patent lacrimal irrigation.

• Partial response = reduction in epiphora by minimum one Munk’s score, delayed FDDT with partially patent lacrimal irrigation.

• Treatment failure = no relief in epiphora (same or worse score on Munk’s grading), positive FDDT and complete block on lacrimal irrigation. 

## Results

A total of 73 eyes of 52 patients were included in the study, with a mean age of 44.5 years. The majority (n=38, 73.1%) of patients were females. Thirty-one (59.6%) patients had unilateral disease, while 21 (40.38%) had bilateral involvement. The mean duration of symptoms before getting the treatment was 22.6 months. The probable etiology of LCO included chronic blepharitis or meibomian gland disease in 32 eyes (43.8%), use of topical prostaglandin eyedrops for glaucoma in 18 (24.6%) eyes, and previous herpes zoster ophthalmicus in 6 (8.2%). Five patients (10 eyes) were using taxanes as chemotherapy. In 3 eyes, *Demodex palpebrum* infestation was noted, while in 4 eyes, no ophthalmic or systemic cause was detected.

 Before presenting to us, 10 patients (16 eyes, 21.9%) had undergone transconjunctival lacrimal gland botulinum toxin injections for epiphora without prolonged satisfactory relief. Lacrimal probing and syringing were done > 3 times in 62 eyes (84.9%) of the patients. The upper LCO or canalicular stenosis was noted in 36 eyes, which was not included in our analysis. The distance of the LCO from the punctum was classified as proximal (< 6mm) and distal (≥ 6mm). The details of LCO (proximal or distal) and Munk’s scoring of patients are compiled in **Table 1**. 

**Table 1:** Patient data of lacrimal canalicular obstructions and treatment outcomes

Type of LCO	Proximal	Type of LCO	Proximal
No. of eyes	38 (52.1%)	35 (47.9%)	73
Preoperative Munk’s score			
≥ 2	2 (5.3%)	2 (5.7%)	4 (5.5%)
3	1 (2.6%)	2 (5.7%)	3 (4.1%)
4	5 (13.1%)	4 (11.4%)	9 (12.3%)
5	30 (78.9%)	27 (77.1%)	57 (78.1%)
Outcomes (response, ≥ 2 criteria)			
*Complete*			
(Munk’s- 0 or 1, FDDT negative, patent irrigation)	7 (18.4%)	19 (54.3%)	26 (35.6%)
*Partial*			
(Munk’s- reduced, FDDT delayed, partial irrigation)	24 (63.2%)	13 (37.1%)	37 (50.7%)
*Failure *			
(Munk’s- same/ increased, FDDT positive, blocked irrigation)	7 (18.4%)	3 (8.6%)	10 (13.7%)
Complications			
• Stent loss	2	1	3
• Stent extrusion	10	2	12
• Granuloma	1	0	1
• Infection	4	1	5
• Ocular irritation	3	4	7

LCT and VAMS were performed in all eyes by a single surgeon (MS) to keep uniformity. After performing the LCT and confirming the patency of the lacrimal drainage system, the dispersive viscoelastic agent was injected in a “string fashion” to facilitate the easier insertion of a 12-14 mm long monocanalicular silicone stent. The patient was asked to look in upgaze and to blink minimally to avoid the viscoelastic loss secondary to the orbicularis oculi muscle contractions. A 10-0 nylon suture was used in 14 eyes (19.2%) due to intraoperative instability of monocanalicular stent as a preventive step to minimize the stent malposition and loss. None reported any viscoelastic-related side-effect or issue in the postoperative period. 

**Fig. 4:**
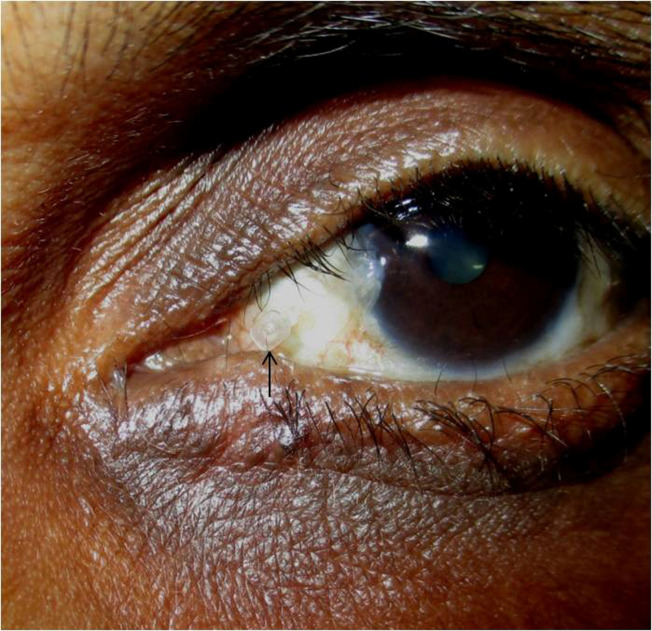
Spontaneous extrusion of the stent with dislocated punctum fixation device

The mean duration of stenting was 13.5 weeks, and in all patients, the monocanalicular stent was kept in-situ for a minimum of 12 weeks. In the postoperative period, 12 (16.4%) eyes needed stent repositioning at a mean follow-up of 1.5 weeks after the procedure. After stent removal, the mean follow-up for all patients was 14.5 months. The FDDT, treatment outcomes, and complications are shown in **Table 1**. Mild punctum discharge with surrounding hyperemia was noted in 12 eyes (16.4%), which were treated with antibiotic ointment (moxifloxacin 0.5%, 2 times/ day x 1 week). Premature stent loss/ displacement (**Fig. 4**) was noted in 7 eyes, in which a new stent was reinserted and kept for the total duration of 12 weeks. Punctum granuloma was noted in 3 patients, of whom 2 responded to topical steroids, while one needed a surgical excision.

## Discussion

The clear fluid epiphora secondary to LCO often reduces the quality of life and causes troublesome visual fluctuations [**1**,**2**]. The patients often seek medical advice for its correction, but the available treatment options are not very popular, even amongst the specialty-trained oculoplastic surgeons. In our study, we reported a long-term satisfactory (complete + partial) response in 86.3% of cases after LCT and VAMS in patients having LCO. The Sisler’s lacrimal trephine provides a novel trephined pathway, recanalizing the obstructed segment of the canaliculus. The injection of viscoelastic agent inside this novel pathway creates a lumen that assists in the easier, smoother, and more efficient insertion of the monocanalicular stent. This step otherwise becomes challenging due to the raw surface and pliable nature of the monocanalicular silicone stent. 

Currently, the management of LCO is broadly focused on improving the tear drainage or reducing tear production. The latter has been advocated by some researchers as a viable treatment option in surgically challenging cases or in which the recanalization procedures have failed. Injection of botulinum toxin (2.5 IU) into the palpebral lobe of the lacrimal gland has shown to provide satisfactory relief in 53-86% of cases [**17**,**18**]. Its described limitations are a restricted duration of action, the theoretical possibility of dry eye, costly and pharmacological side-effects. The lacrimal gland needling has been devised to overcome a few of these limitations and has shown satisfactory results in animal study models [**19**]. However, targeting the physiological production of tears may sometimes lead to dissatisfied patients. Hence, the main emphasis for the treatment of epiphora targets the drainage system.

Sisler’s lacrimal trephine is now an established instrument for the recanalization procedure in patients having LCO at any level (proximal, distal, or common) [**1**-**15**]. The standard operating technique for this mechanical device is now established [**1**,**2**,**8**]. A nasal endoscopic guidance is advised for the post-DCR LCO for best outcomes. Any procedure done under visualization may provide more insights and result in better outcomes. However, the use of transcanalicular dacryoendoscope after LCT has not been reported, probably due to the fresh bleed obscuring the view of the newly trephined tract. 

After LCT, people have used monocanalicular, bicanalicular, double canalicular stenting, and balloon dilatation (ante or retrograde) as an adjunct to keep the newly created pathway patent and functional. Our experience of monocanalicular stents after LCT has been one of the pioneering works in the field [**1**,**8**]. In the present study, we reported a user-friendly innovation of using a viscoelastic agent as an adjunct for the easier insertion of the monocanalicular stents. As the majority of monocanalicular stents (Mini-Monoka®, Aurostent®) do not have a stylet, the use of viscoelastic agents helps in their smoother and faster insertion. 

The literature supports the role of bicanalicular stents, which routinely have a metal bodkin, a helpful adjunct in traversing the trephined portion of the canaliculus. However, their potential chances of punctum cheese-wiring, corneal irritation by stent loop, and handling of unaffected canaliculus and nasolacrimal duct are limiting factors for its use. The use of bicanalicular stents during DCR surgery with canalicular obstructions is reasonably justifiable. **Table 2** mentions the studies featuring the use of lacrimal trephine for canalicular obstructions in various situations. Our study showed a significant success rate (complete + partial) in 86.3% of patients having canalicular obstruction secondary to various etiologies. Once the tear-flow is established over the surface of the stent, the chances of retaining the tract’s patency are considerable due to the riverbed phenomenon.

**Table 2:** Compilation of studies featuring lacrimal canalicular trephination and stenting for canalicular obstructions

Author/ year	No. of patients/ eyes	Pathology (level of canalicular obstruction)	Type of stent used	Stent kept for (months)	Follow-up(months)	Outcomes	Complications
Nathoo et al. [**10**]/ 2013	43/ 45	Post DCR - 32 eyes Common CO - 73% Lower CO - 12% Upper CO - 4% Bicanalicular - 7%	Crawford’s bicanalicular stent	5.6	32.8	Complete - 63% Partial - 25% None - 13%	Repeat intervention - 64%
Zadeng et al. [**8**]/ 2014	23/ 24	Distal lower CO - 100%	Monocanalicular Trephination alone	2	8.6	Complete - 83.3% Partial - 8.3% Failure - 8.3%	Spontaneous stent extrusion - 2
Singh et al. [**1**]/ 2017	32/ 38	Lower proximal CO - 5 Lower distal CO - 21 Common CO - 12	Monocanalicular Trephination alone	1.5- 2	13.5	Complete - 76.3% Partial - 7.8% Failure - 15.8%	Tube extrusion - 4 Conjunctival irritation - 3
Sisler & Allarkhia [**6**]/ 1990	/ 18	CCO	Crawford’s bicanalicular stent	1.5	6-9	Success - 83.3%	Infection - 1 Stent loss - 1
Khoubian et al. [**9**]/ 2006	32/ 41	CCO - 17 Proximal bicanalicular - 11 Distal bicanalicular - 6 Distal LCO - 5	Bicanalicular DCR + trephination	5	12.4	Complete - 49% Partial - 38%	Premature stent removal - 3 Abandoned - 2 Pyogenic granuloma - 1
Paik et al. [**13**]/ 2012	Double - 54/ 58 Single - 50/ 56	Mid-distal - 5 Distal - 21 CCO - 88	Bicanalicular stents (used as single and double)	Double - 4.3 Single - 4.1	Double - 8.7 Single - 8.3	Double - 91.4% Single - 75%	Migration or extrusion - 8 Punctum slit - 2 Canaliculitis - 5 Granuloma - 1
Beak et al. [**20**]/ 2011	29/ 31	Distal CO - 14 Common CO - 17	Bicanalicular stents DCR + trephination	5.7	8.2	Complete - 80.6% Partial - 12.9% Failure - 6.5%	Granuloma around ostium - 26 (83.8%) Septonasal synechiae - 19 (61.3%)
Kong et al. [**15**]/ 2015	57/ 59	Upper CO - 9 Lower CO - 28 Common CO -14 Bicanalicular - 8	Single silicone tube - 25 Double silicone tube - 34 DCR + trephination	4.8	7.8	Upper CO - 66.7% Lower CO - 28 Common CO -14 Bicanalicular - 8	Granulation tissue around osteotomy site - 55.9% Synechiae - 3.3%
Shams et al. [**14**]/ 2016	8/ 8	Common CO - 8	Bicanalicular stents DCR + trephination	3	12	Anatomical - 63% Functional - 63%	No short-term complications

As a measure of postoperative success, the current literature provides evidence about the functional clearance of the fluorescein dye during FDDT. It would be interesting to have a dacryoendoscopic study of the newly created pathway after canalicular trephination and stent removal. Yang et al. (2008) published a series featuring the use of dacryoplasty balloons (2mm) after canalicular trephination in patients with monocanalicular (10 eyes) and common canalicular (56 eyes) obstructions [**21**]. They concluded that balloon dacryoplasty after LCT is a good alternative to CDCR and is a simple and safe technique for canalicular obstructions. However, the use of a lacrimal balloon would add an additional expense to the surgery, which becomes difficult to afford for the general public of developing nations. Hence, our indigenous technique of VAMS provides a satisfactory success rate in the majority of the patients. 

## Conclusion

In conclusion, LCT with VAMS insertion provides a safe, effective, and successful treatment modality for the management of epiphora secondary to canalicular obstructions. We advocate using a viscoelastic agent for easier, faster, and successful insertion of monocanalicular stent via the trephined canalicular tissues. 

**Conflict of Interest statement**

The authors state no conflict of interest.

**Informed Consent and Human and Animal Rights statement**

Informed consent has been obtained from all individuals included in this study.

**Authorization for the use of human subjects**

Ethical approval: The research related to human use complies with all the relevant national regulations, institutional policies, is in accordance with the tenets of the Helsinki Declaration, and has been approved by the review board of Advanced Eye Centre, Post Graduate Institute of Medical Education and Research, Chandigarh, India.

**Acknowledgments**

None.

**Sources of Funding**

None.

**Disclosures**

None.
